# Transcriptome Profile of Yeast Strain Used for Biological Wine Aging Revealed Dynamic Changes of Gene Expression in Course of Flor Development

**DOI:** 10.3389/fmicb.2020.00538

**Published:** 2020-04-03

**Authors:** Andrey V. Mardanov, Mikhail A. Eldarov, Alexey V. Beletsky, Tatiana N. Tanashchuk, Svetlana A. Kishkovskaya, Nikolai V. Ravin

**Affiliations:** ^1^Institute of Bioengineering, Research Center of Biotechnology of the Russian Academy of Sciences, Moscow, Russia; ^2^Research Institute of Viticulture and Winemaking “Magarach” of the Russian Academy of Sciences, Yalta, Russia

**Keywords:** *Saccharomyces cerevisiae*, flor yeast, sherry, genetic diversity, comparative genomics, biofilm, SNP

## Abstract

Flor strains of *Saccharomyces cerevisiae* are principal microbial agents responsible for biological wine aging used for production of sherry-like wines. The flor yeast velum formed on the surface of fortified fermented must is a major adaptive and technological characteristic of flor yeasts that helps them to withstanding stressful winemaking conditions and ensures specific biochemical and sensory oxidative alterations typical for sherry wines. We have applied RNAseq technology for transcriptome analysis of an industrial flor yeast strain at different steps of velum development over 71 days under experimental winemaking conditions. Velum growth and maturation was accompanied by accumulation of aldehydes and acetales. We have identified 1490 differentially expressed genes including 816 genes upregulated and 674 downregulated more than 2-fold at mature biofilm stage as compared to the early biofilm. Distinct expression patterns of genes involved in carbon and nitrogen metabolism, respiration, cell cycle, DNA repair, cell adhesion, response to various stresses were observed. Many genes involved in response to different stresses, oxidative carbon metabolism, high affinity transport of sugars, glycerol utilization, sulfur metabolism, protein quality control and recycling, cell wall biogenesis, apoptosis were induced at the mature biofilm stage. Strong upregulation was observed for *FLO11* flocculin while expression of other flocculins remained unaltered or moderately downregulated. Downregulated genes included those for proteins involved in glycolysis, transportation of ions, metals, aminoacids, sugars, indicating repression of some major transport and metabolic process at the mature biofilm stage. Presented results are important for in-depth understanding of cell response elicited by velum formation and sherry wine manufacturing conditions, and for the comprehension of relevant regulatory mechanisms. Such knowledge may help to better understand the molecular mechanisms that flor yeasts use to adapt to winemaking environments, establish the functions of previously uncharacterized genes, improve the technology of sherry- wine production, and find target genes for strain improvement.

## Introduction

Biological wine aging is a multistep technological process used in several countries for production of sherry type wines. The essence of this process is the use of a special race of *Saccharomyces cerevisiae*, the flor yeast that are responsible for the majority of biochemical changes affecting composition and sensory properties of these wines. Biochemical and physiological conditions of biological wine aging are different from those in the course of fermentation. Flor yeast strains that form a biofilm on the surface of fortified fermented must display drastic alterations in carbon and nitrogen metabolism. In the absence of fermentable sugars flor yeast use ethanol and glycerol as major carbon sources through oxidative catabolism. They shift to use poor nitrogen sources and withstand harsh and stressful conditions of biological wine aging with high ethanol and acetaldehyde concentrations, oxidative damage and limitations of many essential nutrients (Alexandre, 2013; Legras et al., 2016). Velum formation is considered to be a major adaptive property of flor yeast ensuring oxygen access and protecting them from various stresses (Esteve-Zarzoso et al., 2001).

Specific phenotypic and biochemical properties of flor yeast are thought to be the result of domestication and artificial selection that were fixed in the course of separation of flor yeast as a sister branch from Wine/European clade (Legras et al., 2014). Recent extensive comparative genomic analysis supports and extends this view. Comparison of wine and flor yeast genomes have revealed a complex landscape of genetic variation specific for flor yeast including hundreds of SNPs, InDels, chromosomal rearrangements, gene content alterations (Coi et al., 2017; Eldarov et al., 2018; Legras et al., 2018). Many of these structural variations have affected key regulatory molecules (transcription factors, signal transduction molecules), suggesting direct influence on gene expression patterns of target genes, involved in metabolic and morphological changes specific for flor yeast (Coi et al., 2017; Eldarov et al., 2018).

Comparative transcriptome analysis is a powerful tool for the study of global dynamics of yeast gene expression in response to various environmental, genetics and chemicals cues. In recent years hundreds of transcriptome studies performed either with microarray or RNAseq platforms provided enormous resource for understanding the whole-genome regulatory networks and mechanisms of yeast gene expression in response to various stresses (Taymaz-Nikerel et al., 2016). While majority of these reports use laboratory strains and artificial conditions, several directly address the dynamics of gene expression in industrial conditions related to wine, beer, and bioethanol production. In one of the first studies significant alterations in the expression of more than 2000 genes were detected in the course of adaptation of wine yeast strain EC1118 to changing physiological and biochemical conditions of wine fermentation (Rossignol et al., 2003). Genes involved in many metabolic and signaling pathways underwent coordinated regulation, including genes associated with wine fermentation, genes involved in nitrogen catabolism and others. Under conditions of low-temperature fermentation, important to improve wine quality, cold shock genes, genes involved in cell cycle progression and cell proliferation were upregulated (Beltran et al., 2006). These and other transcriptional changes correlated with increased cell viability, improved ethanol tolerance, increased production of short chain fatty acids and esters (Tronchoni et al., 2017). Significant inter-strain differences in the expression patterns of about 30% of genes responsible for aroma formation were detected in 3 yeast strains in the course of wine fermentation at normal (28°C) and low (12°C) temperatures (Gamero et al., 2014). Notably, altered expression was observed for many genes important for winemaking, encoding acyl acetyltransferases, decarboxylases, aldehyde dehydrogenases, alcohol dehydrogenases, enzymes involved in amino acid transport and metabolism. This and many other studies, in particular for the analysis of gene expression in wine yeast strains depending from the availability of nitrogen sources (Rossignol et al., 2003; Barbosa et al., 2015) clearly show that variations in gene expression is closely associated with phenotypic variation of wine strains (Salvado et al., 2008; Walker et al., 2014; García-Ríos and Guillamón, 2019).

Significant changes in wine yeast transcriptome occur during the transition to the stationary phase. Thus, the 223 genes dramatically induced at different stages of fermentation have been allocated to a specific group of Fermentation stress response (FSR) genes, wherein more than 60% of these genes has previously unknown functions (Marks et al., 2008).

Studies of differential protein expression in the course of sherry-wine formation is limited, however recently several reports documenting semi-quantitative proteomic analysis of cytosolic, cell-wall and mitochondrial proteins of industrial flor strain under laboratory “non-biofilm” and “biofilm” conditions have been carried out (Moreno-García et al., 2015, 2016, 2017, 2018a). These studies revealed alterations in the abundance of proteins involved in respiration, translation, stress damage prevention, DNA reparation, carbon and amino acid metabolism, some interesting finding associated with the changes in abundance of flor yeast enzymes affecting sensory properties of sherry wine.

Here, we report a comprehensive transcriptome analysis of *flor yeast gene expression* using RNA-seq data. We generated transcriptomic data for a recently sequenced flor yeast strain I-329, for which detailed microbiological, biochemical, physiological data is also available (Kishkovskaia et al., 2017; Eldarov et al., 2018). This strain was grown under conditions mimicking biological wine aging, at three consecutive stages of velum formation and growth. This dataset was used to determine the expression levels of each of the genes at different stages of velum formation, to reveal novel regulatory networks and expression patterns, including those for previously uncharacterized genes. We observed distinct expression patterns of genes involved in carbon and nitrogen metabolism, respiration, cell cycle, DNA repair, cell adhesion, response to various stresses etc., illuminating the complex landscape of gene expression associated with specific oenological conditions. These data will help to better understand flor yeast physiology and adaptive evolutionary changes pertinent to their oenological properties, establish the functions of previously uncharacterized yeast genes, improve the technology of sherry wine production, find target genes for subsequent strain improvement programs.

## Materials and Methods

### Cultivation of Flor Yeast and Sampling

The strain used was *S. cerevisiae* I-329, industrial flor yeast from the culture collection of the Research Institute of Viticulture and Winemaking “Magarach” of the Russian Academy of Sciences (Kishkovskaia et al., 2017).

Sterilized Albillo grape must (concentration of sugars 230 g/l, concentration of titratable acids 5.4 g/l, pH 3.5) was inoculated with a pure culture of strain I-329 with a seed dose of 2 × 10^6^ cells/ml and fermented for 28 days at room temperature in a 3 l glass balloon. At the end of fermentation the wine was fortified to 14.5% ethanol and incubation was continued. The firm yeast velum, covering about 90% of the entire surface became formed 10 days after adding alcohol (Supplementary Figure S1). At this point the first sample of floating flor (“early biofilm,” Table 1) was taken. 45 days later completion of continuous film growth was observed and at this point the second sample (“thin biofilm,” Supplementary Figure S1) was taken. The third, “mature biofilm,” sample was taken 26 days after the second sample, at this point the mature folded biofilm (Supplementary Figure S1) was formed (Table 1). Sampling at each time point was carried out in triplicate. Samples were taken with a small spoon at three points of the floating biofilm covering the surface of the balloon (pieces about 2–5 cm in diameter weighting 100–300 mg). The samples included the entire film thickness, from the upper layer exposed in air to the lower layer in contact with the liquid. Biofilm samples were immediately placed in the RNA later solution (Invitrogen, United States) and kept at –20°C until RNA isolation.

**TABLE 1:** Chemical composition of wine samples.

**Sample designation**	**Early biofilm**	**Thin biofilm**	**Mature biofilm**
Days from inoculation to sampling	38	83	109
Ethanol% (v/v)	12.4	10.8	9.6
Volatile acidity (g/l)*	0.2	0.2	0.1
Total acidity (g/l)**	7.8	7.4	7.0
Aldehydes (mg/l)	382.8	531.3	668.8
Acetals (mg/l)	147.2	253.7	280.3
pH	3.6	3.6	3.6
Glucose (g/l)	0.2	0.1	<0.1
Fructose (g/l)	<0.1	<0.1	<0.1
Oligosaccharides (g/l)	0.3	0.2	0.2
Glycerol (g/l)	8.5	7.9	6.8

*Expressed in grams of tartaric acid per liter. **Expressed in grams of acetic acid per liter.

### Chemical Analysis of Wine Samples

At the time of sampling aliquots of wine were also taken for chemical analyzes. The content of volatile and titratable acids, alcohol, aldehydes and acetals was determined using standard methods adopted in winemaking (Compendium of international methods of wine and must analysis, 2018) and as described earlier (Kishkovskaia et al., 2017).

Ethanol content was measured with a hydrometer. Total acidity was measured by titration with bromothymol blue as indicator and comparison with an end-point color standard and expressed in grams of tartaric acid. Volatile acids, derived from the acids of the acetic series, were separated from the wine by steam distillation and titrated using standard sodium hydroxide and expressed in grams of acetic acid per liter. The mass concentration of aldehydes was determined by a method based on the ability of aldehydes to bind to sodium hydrosulfite in a complex non-volatile compound. Excess of hydrosulfite was oxidized with iodine and then the aldehyde sulfite compound was decomposed with alkali. The liberated sulfur aldehyde was titrated with a 0.005 M iodine solution. Acetaldehyde accounted for more than 90% of all aldehydes. The mass concentration of acetals was determined by the method based on vacuum distillation of aldehydes and volatile acetals of wine with further acid cleavage of the acetals remaining in the flask and determination of the released volatile aldehydes by iodometric titration.

Glucose, fructose, oligosaccharides, and glycerol were determined by liquid chromatography with a Shimadzu LC-20AD chromatograph (Shimadzu, Japan).

### Transcriptome Sequencing and Analysis of Gene Expression

Nine RNA samples (three sampling points, each in three replications) were used for transcriptome analysis of strain I-329 in course of flor maturation. The total RNA was extracted employing a hot phenol method (Schmitt et al., 1990) followed by purification using RNeasy Mini Kit (Qiagen GmbH, Germany). mRNA library preparation was performed using an NEBNext mRNA Library Prep Reagent Set for Illumina according to the manufacturer’s instructions (New England Bio-Labs Inc., Ipswich, MA, United States). The libraries were sequenced using the Illumina HiSeq 2500 platform (Illumina, San Diego, CA, United States). Six to nine million of 50-bp single-end reads were generated for each sample. The previously assembled genome of *S. cerevisiae* strain I-329 (GenBank PTER00000000, Eldarov et al., 2018) was used as a reference for the expression analysis.

Gene expression levels were estimated using RSEM v.1.3.1 software (Li and Dewey, 2011) and recorded in reads per kilobase per million mapped reads (RPKM). Mapping of RNA-seq reads to genes (excluding introns) was performed using program Bowtie2 (Langmead and Salzberg, 2012), with parameters selected by RSEM script. Statistical analysis of differential expression was performed using EBSeq v. 1.2.0 package (Leng and Kendziorski, 2019), invoked by RSEM script. To quantify evidence in favor of differential expression EBSeq provide posterior probabilities of differential expression (PPDE), PPDE values above 0.95 were considered as statistically significant. Note that EBSeq is more conservative method for identification of differentially expressed genes than DESeq program (Anders and Huber, 2010) which provides *P-*values adjusted for multiple testing using Benjamini–Hochberg method (Leng et al., 2013).

For the analysis of gene expression in flor yeast strain CECT10094 (the dataset obtained on AB SOLiD 4 System, retrieved from SRR9027083; Ibáñez et al., 2017) we used SHRiMP v.2.2.3 (Rumble et al., 2009) to map colorspace. The reads were mapped on strain I-329 genome and RSEM v.1.3.1 software was used for the following expression analysis.

Volcano plots were generated using custom perl and R scripts, using RSEM results as an input. To visualize expression on KEGG pathways we used Pathview and KEGGREST Bioconductor v.3.8 packages run with custom scripts in R environment v.3.6.0 (Luo and Brouwer, 2013; Tenenbaum, 2019).

### Gene Ontology Functional Categories Analysis

Gene ontology (GO) enrichment analysis of differentially expressed genes was performed using PANTHER database (Mi et al., 2019). Fischer’s exact test with Bonferroni correction and a *p*-value < 0.05 were used to filter the results. For submission to database gene names were converted from LOCUS_TAGs to UNIPROT_IDs using online DAVID Gene ID Conversion Tool v.6.8 (Jiao et al., 2012) available at https://david.ncifcrf.gov/conversion.jsp. The concise list of terms was obtained after removing redundant GO terms with the help of ReViGo web tool (Supek et al., 2011). Search and analysis of transcription factors (TF) and TF-binding sites was done using the Yeasttract database (Teixeira et al., 2018).

### Nucleotide Sequence Accession Numbers

This BioProject has been deposited in GenBank under accession number PRJNA592304. The sequences obtained in this project have been deposited in the NCBI Sequence Read Archive under the accession numbers SRR10551657-SRR10551665 (Supplementary Table S1).

## Results

### Changes of Composition of Fortified Fermented Must in Course of Flor Growth

To gain insight into the dynamics of transcriptional responses of industrial flor yeast strain at stage of biofilm formation we used the previously sequenced flor strain I-329 (Eldarov et al., 2018). This strain was chosen because it is used for industrial production of sherry-like wine in Russia, it is tolerant to high ethanol concentration (16–17%) and could retain high viability at the biofilm stage (Kishkovskaia et al., 2017).

The strain I-329 was used to ferment the must and upon completion of fermentation the must was fortified by addition of ethanol to 14.5%. In course of further incubation the yeasts appeared on the surface and 10 days after ethanol addition formed a tiny biofilm covering about 90% of the surface of the balloon. At this point the first biofilm sample (early biofilm) was taken. The second sample (thin biofilm) was collected when the flor covered the whole surface of the balloon, and the third sample was obtained after formation of mature biofilm (Table 1). Throughout all three stages chemical composition of wine was monitored (Table 1). In the course of velum growth volumetric ethanol concentration dropped from 12.4% to 9.6%, while the concentration of aldehydes and acetals almost doubled reaching, respectively, 668.8 mg/l and 280.3 mg/l, illustrating the typical dynamics of ethanol oxidation by yeast during biofilm maturation.

### Global Transcriptome Variation at Different Steps of Flor Development

For nine samples a total of 64 million reads were obtained with at least 5.9 million reads for each sample (Supplementary Table S1). Of 5323 protein-coding genes annotated in the nuclear genome, 5320 genes were expressed in at least one sample. Analysis of RNA-seq data revealed a global gene expression profile of I-329 strain in the course of flor biofilm maturation (Supplementary Table S2).

**FIGURE 1:**
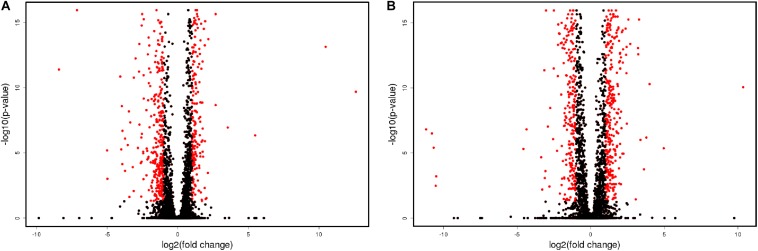
Volcanic plot representation of changes in gene expression. Genes up-regulated or down-regulated more than 2-fold are shown in red. **(A)** Sample 2 vs. sample 1; **(B)** sample 3 vs. sample 1.

Volcanic plot shows that in the process of flor growth there was a notable increase in the amount of differentially expressed genes (DEGs) upregulated or downregulated in the course of flor growth as compared to the early biofilm stage (Figure 1). In total we have identified 1490 DEGs with more than 2-fold statistically significant (PPDE > 0.95) difference of expression level between the mature biofilm stage and the early stage. Of these 565 genes were upregulated 2–4 fold (Up2), and 528 genes (Dn2) showed 2–4 fold decline in the expression level (Table 2). A group of 397 DEGs with more pronounced differences in gene expression levels included 251 genes upregulated more than 4-fold (Up4) and 146 genes downregulated more than 4-fold (Dn4) at the mature biofilm stage as compared to the early stage. Five genes, namely *YJL218W* (acetyltransferase), *AIF1* (apoptosis-inducing factor 1), *ENB1* (siderophore iron transporter), *REE1* (regulator of enolase expression), *CSS3* (hypothetical protein) were found to be expressed only at the mature biofilm stage.

**TABLE 2:** Numbers of up- and down-regulated genes during flor development.

**Expression pattern**	**Set**	**Regulation range**	**Thin biofilm vs. early biofilm**		**Mature biofilm vs. thin biofilm**		**Mature biofilm vs. early biofilm**	
			**All genes**	Genes containing flor yeast specific SNPs*	**All genes**	Genes containing flor yeast specific SNPs*	**All genes**	Genes containing flor yeast specific SNPs*
Up-regulated	Up4	X ≥ 4	84	21	122	34	251	55
	Up2	4 > X ≥ 2	398	81	458	121	565	133
Down-regulated	Dn2	0.25 < X ≤ 0.5	355	105	340	85	528	148
	Dn4	X ≤ 0.25	66	19	52	10	146	33
Total DEG			903	226	972	250	1490	369

*1334 protein-coding genes containing flor-yeast specific SNPs (Eldarov et al., 2018).

GO analysis was performed for these four gene sets. For the Up2 set there was notable diversity of enriched terms in all 3 GO categories (Supplementary Table S3). The most prevalent in the “Biological process” category were such terms as “cell-redox homeostasis,” “mitochondrial respiratory chain complex assembly,” various terms related to mitochondrial protein synthesis, proteasome-mediated protein degradation etc. Gene products of these DEGs were predicted to have mitochondrial and proteasomal localization (“TCA complex,” “mitochondrial ribosome,” “proteasome complex”) with “molecular functions” related to “structural constituent of ribosome” and “structural molecule activity” (Supplementary Table S3). For the Up4 set enriched the “Biological Process” terms were those related to sulfur metabolism, biosynthesis of sulfur-containing aminoacids, protein refolding etc. In the “Cellular Component” and “Molecular Function” categories the number of enriched terms was much smaller and limited to “mitochondrial intermembrane space,” “organelle envelope lumen” and “unfolded protein binding” (Supplementary Table S4).

The Dn2 group of 528 moderately downregulated DEGs (Supplementary Table S5) was enriched in genes for proteins with organelle membrane localization, with functions related to binding and transportation of ions, metals and small molecules. In the “Biological Process” category overrepresented were terms related to “regulation of transcription from RNA polymerase II promoter in response to oxidative stress,” “glycolytic process,” “ATP generation from ADP,” etc. Altogether these data indicates downregulation of several transport and metabolic process at the mature biofilm stage. This trend was more evident in the Dn4 group of 146 DEGs (Supplementary Table S6). This group includes genes for proteins involved in metabolism of nucleotides and sugars. In particular, strong downregulation was observed for low affinity glucose transporter *HXT1* (150-fold), *OPT1* oligopeptide transporter (40-fold), *TPO4* and *VBA1* polyamine and general amino acid transporters (∼16-fold), some enzymes involved in glycolysis (*PGK1, ENO2, TDH1*).

Differentially expressed genes within the four sets (Up2, Up4, Dn2, Dn4) represented a wide range of KEGG functional categories. To gain more detailed insight in the nature of transcriptional responses in the course of growth and maturation of flor yeast velum we have analyzed expression levels of genes assigned to various functional KEGG groups and categories.

**FIGURE 2:**
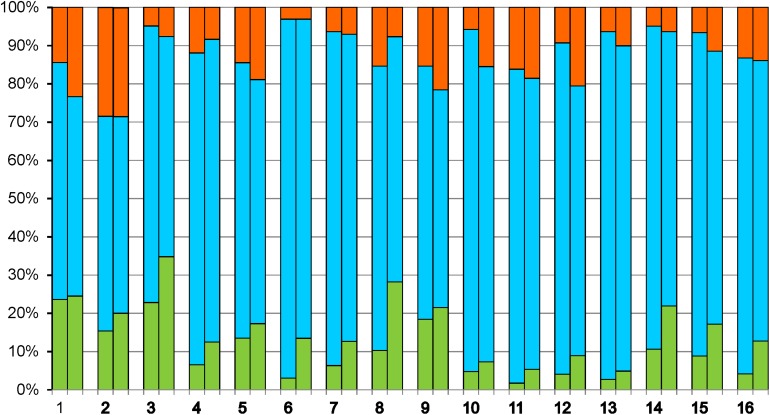
Up-regulation and down-regulation of genes distributed to various KEGG categories. Metabolism: 1 – Carbohydrate metabolism, 2 – Energy metabolism, 3 – Lipid metabolism, 4 – Nucleotide metabolism, 5 – Amino acid metabolism, 6 – Glycan biosynthesis and metabolism, 7 – Metabolism of cofactors and vitamins, 8 – Biosynthesis of other secondary metabolites, 9 – Xenobiotic biodegradation and metabolism. Genetic Information Processing: 10 – Transcription, 11 – Translation, 12 – Folding, sorting and degradation, 13 – Replication and repair. Environmental Information Processing: 14 – Signal transduction. Cellular Processes: 15 – Transport and catabolism, 16 – Cell growth and death. Fractions of up-regulated genes are shown in red, down-regulated in green, and genes without significant changes in expression in blue. In each category, the left and right parts of the column indicate the changes of gene expression in the second and in the third sample, respectively, relative to the first sample.

KEGG pathway analysis showed major alterations in expression levels of genes belonging to the categories of “Energy metabolism” and “Carbohydrate metabolism,” reflecting changes in yeast metabolism during biofilm growth as related to exhaustion of sugars and shift to ethanol oxidation (Figure 2).

For the vast majority of DEGs, a unidirectional trend (up- or downregulation) in the expression level was observed from the early biofilm stage to the thin biofilm, and further to the mature biofilm stage. Nevertheless, a small group of genes had a maximum (64 genes) or a minimum (87 genes) of expression level at the second stage, which was more than two times different from the expression levels in both the early and mature biofilm stages (Supplementary Table S7). Therefore, in the following sections we compared the expression levels in the first and the last flor samples; genes most activated or repressed at the thin biofilm stage are described in the last section of the “Results.”

### Transcription Profiles of Genes Carrying Flor Yeast Specific SNPs

Previous taxonomic and comparative genomic studies of wine and flor yeast strains showed that flor yeasts form a separate phylogenetic group artificially selected in the course of domestication and possessing unique physiological and genetic properties (Legras et al., 2014; Coi et al., 2017; Eldarov et al., 2018). Genome-wide comparative analysis of wine and flor strains revealed 2270 flor-yeast specific SNPs located in 1337 loci (Eldarov et al., 2018) with 73 SNP located in intergenic regions and the rest in 1334 protein-coding genes. Transcriptome analysis of these genes revealed 369 DEGs (27.7%) with more than 2-fold alterations in expression levels, 188 upregulated and 181 downregulated (Table 2). Since the shares of up- and downregulated genes were nearly the same for the whole genome (28%), these data did not support the hypothesis that genes carrying flor-specific SNPs should be preferentially differently expressed in course of flor growth.

### Top Highly Expressed Genes

Quantification of the expression levels of analyzed DEGs enabled to select groups of highly expressed genes at the early biofilm and mature biofilm stages. Superposition of the lists of top 250 genes with highest expression level at these two stages revealed a group of 121 common highly expressed genes and genes, specifically expressed at one or another stage (Supplementary Table S8).

The list of 121 common highly expressed genes is a mixed collection of those for some heat-shock proteins, components of mitochondrial electron transfer chain and ATP generation machinery, enzymes of glycolytic pathway and sugar transporters, transcription and translation elongation factors etc. Despite relatively high expression level at two stages many genes showed notable alteration in transcription. For instance, genes for many HSP proteins showed 5–10 fold upregulation at the mature biofilm stage, genes involved in oxidative phosphorylation were upregulated 2–4 fold at this stage, while strong downregulation was observed for genes for glycolytic enzymes, plasma membrane ATPase gene *PMA1*, and low affinity glucose transporter gene *HXT3* (Supplementary Table S8).

We have also compared the list of 121 genes highly expressed at both early and mature biofilm stages and the list of top 250 genes highly expressed during alcoholic fermentation using the recently published RNA-seq data obtained for flor yeast strain CECT10094 after 7 days of fermentation in synthetic must media (Ibáñez et al., 2017). We found 79 common genes between these sets (Supplementary Table S9). This common set included genes for glycolysis and gluconeogenesis, transport of sugars, glycogen and trehalose metabolism, heat-shock and oxidative stress response, ATP generation machinery. It is interesting to note that the *YML131W* gene, which encodes a protein with an unknown function, has a constantly high expression level both under fermentation conditions and in biofilm-grown cells. According to SGD description^1^ this protein is similar to medium chain dehydrogenase/reductases and was induced by various stresses including osmotic shock, DNA damaging agents, and other chemicals.

### Possible Transcriptional Control of Differentially Expressed Genes

Numerous data concerning transcriptional responses of yeast cells to different environmental perturbations revealed the key role of stress-responsive transcriptional factors (TFs) in sensing and protection against environmental damage (Causton et al., 2001; Roberts and Hudson, 2006; Berry and Gasch, 2008; Taymaz-Nikerel et al., 2016). Using the data available at YeasTract database we have counted TF-binding sites in the promoters of highly upregulated (Up4) and downregulated (Dn4) genes and selected TF with most frequent sites (Supplementary Table S10). Most numerous in the promoters of Up4 genes were sites for TFs with established role in regulating stress-responsive genes (Cin5, Msn2, Msn4, Hsf1, Yap1), TF regulating genes for carbon and nitrogen utilization (Adr1, Gcn4), control of cellular morphogenesis (Fkh1, Tec1, Ste12), confirming the role of these TF and their targets in stress resistance and metabolic and morphological changes of yeast cells specific to biofilm stage.

The genes for selected TF showed variable patterns of regulation, but majority of TF genes showed induction at the mature biofilm stage, with the highest upregulation level observed for *SWI5* (about 30-fold induction) coding for TF binding at stress response elements of responsive genes and for *MET4, MET28, MET32* regulating biosynthesis of sulfur-containing aminoacids. *MSN2* and *MSN4* were downregulated at the mature biofilm stage (Supplementary Table S10).

This TF set partially overlapped with the list of TF identified as possessing most frequent sites in the promoters of strongly downregulated genes (Supplementary Table S10). Msn2, Rap1, Ste12, Sok2, Yap1 sites were among the most frequent. Specific for this set were sites for Gln3, Hap1, Ino4, Pdr1, Skn7 and Yox1 regulating amino acid biosynthesis, oxidative stress response, regulation of cell cycle, and pseudohyphal growth.

### Carbohydrates and Energy Metabolism

**FIGURE 3:**
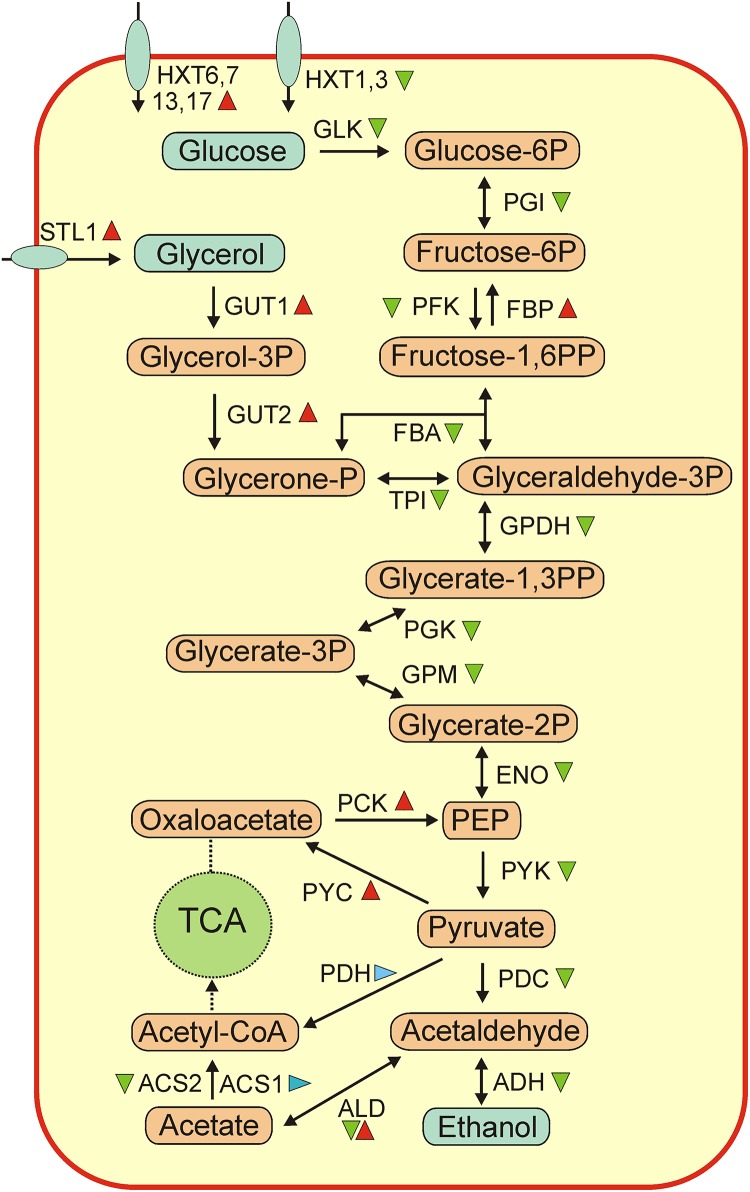
An overview of changes of expression of genes involved in central metabolic pathways. Abbreviations: GLK, glucokinase; PGI, glucose-6-phosphate isomerase; PFK, 6-phosphofructokinase; FBP, fructose 1,6-bisphosphate 1-phosphatase; FBA, fructose-bisphosphate aldolase; TPI, triose-phosphate isomerase; GPDH, glyceraldehyde-3-phosphate dehydrogenase; PGK, phosphoglycerate kinase; GPM, phosphoglycerate mutase; ENO, enolase; PCK, phosphoenolpyruvate carboxykinase; PYK, pyruvate kinase; PYC, pyruvate carboxylase; PDH, pyruvate dehydrogenase; PDC, pyruvate decarboxylase; ADH, alcohol dehydrogenase; ALS, aldehyde dehydrogenase; ACS, acetate- CoA ligase; TCA, tricarboxylic acids cycle; STL1, glycerol proton symporter; HXT, hexose transporter. Genes, up-regulated more than 2-fold at the mature biofilm stage relative to the early biofilm are marker by red triangles, ones down-regulated more than 2-fold, – by green triangles, and ones without significant changes in expression, – by blue triangles. Note that most of ALD genes were up-regulated (*ALD 1, 3, 4, 5*) while *ALD2* was down-regulated.

According to KEGG functional analysis most notable transcriptional changes during flor yeast biofilm development were observed for genes belonging to the categories of “Carbohydrate Metabolism” and “Energy Metabolism.” Glycolysis is the central route of carbohydrate metabolism in yeast. During velum development expression levels of almost all glycolytic genes (*HXK2, PGI1, PFK1, FBA1, TPI1, TDH1, TDH2, PGK1, GPM1, GPM2, ENO1, ENO2, PYK2*, and *CDC19)* decreased more than 2-fold (Figure 3 and Supplementary Table S11). This trend reflected an exhaustion of glucose in the growth medium (Table 1) and a switch of flor yeasts to other carbon sources.

Contrary to decreased transcription of the majority of glycolytic genes, expression of key genes involved in gluconeogenesis - phosphoenolpyruvate carboxykinase (*PCK1*) and fructose-1,6-bisphosphatase (*FBP1*) increased significantly (Supplementary Table S11). Apparently, their induction is required for biosynthesis of sugars under conditions of glucose exhaustion.

The transcription of most genes of the pentose-phosphate pathway (PPP) was not changed significantly, except that for 6-phosphogluconate dehydrogenase (*GND1* and *GND2*), transketolase (*TKL2*), and glucose-6-phosphate dehydrogenase (*ZWF1*). Levels of *GND1* mRNA decreased more than 5-fold at the mature biofilm stage. This data corresponds to previous observation (Zhang et al., 2004), showing a decrease in *GND1* expression upon growth on ethanol and lactic acid as carbon sources. Significantly increased were expression levels of *ZWF1* and *TKL2* genes. *TKL2* upregulation may be related to known patterns of induction of this gene in carbon-limited cultures and in the course of diauxic shift (Boer et al., 2003). *ZWF1* is the first PPP enzyme, catalyzing the rate-limiting and irreversible step (Nogae and Johnston, 1990). *ZWF1* is known to be important for adaptation to oxidative stress (Juhnke et al., 1996).

Expression changes of genes for key pyruvate metabolic enzymes were variable. While the expression of pyruvate carboxylases (*PYC1* and *PYC2*) more than doubled, the expression of pyruvate decarboxylases (*PDC1, PDC5, PDC6*) decreases several fold. *PYC1* and *PYC2* convert pyruvate to oxaloacetate, the substrate important for anaplerosis and gluconeogenesis (Pronk et al., 1996). Pyruvate decarboxylases are key enzymes for alcoholic fermentation, degrading pyruvate to acetaldehyde and carbon dioxide, and also important for oxidation of other 2-oxo acids (Flikweert et al., 1996). Downregulation of these genes likely reflected suppression of fermentative metabolism in course of biofilm growth. Likewise, strong down-regulation was observed for alcohol dehydrogenase *ADH1*, fermentative isozyme, required for the reduction of acetaldehyde to ethanol. The switch of yeasts to ethanol consumption was also reflected by strong up-regulation of aldehyde dehydrogenases (*ALD4, ALD5, ALD6*), required for growth on ethanol and conversion of acetaldehyde to acetate. No significant changes of expression were observed for *ACS1* gene coding for acetate-CoA ligase 1, known to be expressed during growth on non-fermentable carbon sources and under aerobic conditions, while *ACS2* gene coding for the acetate-CoA ligase 2 required for growth on glucose under anaerobic conditions was downregulated.

Under aerobic conditions *S. cerevisiae* can use glycerol as a sole carbon and energy source. At the mature biofilm stage two key genes for glycerol catabolism, glycerol kinase *GUT1* and glycerol-3-phosphate dehydrogenase *GUT2*, were induced more than 8-fold. These genes are known to be repressed when cells are grown on fermentable carbon sources and upregulated on non-fermentable carbon sources such as glycerol or ethanol (Sprague and Cronan, 1977; Grauslund and Rønnow, 2000). Gene *STL1* encoding glycerol proton symporter was upregulated more than 5-fold. Probably, upon exhaustion of glucose in the medium the yeasts switched to the use of remaining glycerol. This is consistent with the observed decrease in glycerol concentration in wine as the velum grew (Table 1).

Most genes for mitochondrially located TCA enzymes showed increased expression at the mature biofilm stage, while paralogous genes for proteins with cytosolic and peroxisomal localization involved in other pathways showed decreased expression, as seen, for instance for mitochondrial (*MDH1*) and cytoplasmic (*MDH2*) malate dehydrogenases.

Expression of genes relevant to the electron transport chain shows different dynamics. Moderate up-regulation was observed for genes coding for subunits of the mitochondrial F_1_F_0_ ATP synthase, the ubiquinol cytochrome c reductase, and the NADH-ubiquinone reductase, while no clear trend was observed for cytochrome c oxidase and succinate dehydrogenase subunits (Supplementary Table S11).

### DNA Repair

It is generally supposed that under biological wine aging conditions flor yeast experience chronic genotoxic stress due to mutagenic action of elevated ethanol and acetaldehyde concentrations. Concentration of aldehydes considerably increased in course of biofilm growth while ethanol concentration moderately decreased (Table 1). Acetaldehyde can form DNA-protein and DNA–DNA crosslinks and adducts that interfere with DNA replication. As shown in recent comprehensive study in fission yeast, acetaldehyde causes a variety of DNA damage and induces different DNA repair pathways (Noguchi et al., 2017). Comparison of mRNA levels of DNA repair genes at the early and mature biofilm stages showed mild induction of some nucleotide excision repair (NER) and mismatch repair (MMR) genes at mature biofilm stage, suggesting that cell has already adapted to this type of DNA damage in the beginning of the experiment (Supplementary Table S12). More notable induction was observed for the majority of genes involved in homologous recombination, highest upregulation observed for *RAD59, RAD54, RDH54*, involved in DNA double strand break repair and overcoming barriers to DNA replication fork progression due to DNA damage. Perhaps, elevation of concentration of acetaldehyde in course of flor maturation induces this type of DNA damage thus activating homologous recombination pathway.

### Cell Wall Structure and Biogenesis

Biofilm formation of flor yeast on the surface of fortified wine materials is a critical prerequisite for efficient biological wine aging. Fungal biofilms are complex structures composed of cells and extracellular matrix. Genetic control of yeast biofilm formation is best studied in *Candida albicans*, and capacity for biofilm formation is directly linked to virulence in this human pathogen (Chandra et al., 2001). Surprisingly, the data about genetic and biochemical control of biofilm formation in yeast are rather limited. The involvement of small heat-shock protein Hsp12 (Zara et al., 2002), v-snare protein Btn2 (Espinazo-Romeu et al., 2008), as well as the dominant role of Flo11 cell surface adhesion molecule for biofilm formation was established using gene knockout and/or overexpression approaches (Fidalgo et al., 2006; Brückner et al., 2012). Recent proteomic study compared abundance of various groups of proteins in flor yeast at biofilm and non-biofilm stage. Notable overrepresentation of several cell wall proteins at the biofilm stage was detected (Moreno-García et al., 2017). The role of some of identified cell surface proteins (Ccw14, Ygp1) in velum formation was confirmed in subsequent gene-knockout experiments with haploid flor yeast strain (Moreno-García et al., 2018b).

We have compared expression levels of the genes for 60 proteins known as cell wall components and enzymes involved in cell wall modification (Supplementary Table S13). The most significant upregulation at the mature biofilm stage relative to the early biofilm stage was observed for *FLO11* (39-fold induction). The genes for other structural GPI-anchored PIR-proteins were also significantly induced, including *CIS3* (19-fold), *PIR1* (15-fold), *PIR3* (3-fold), *SRL1*(14-fold). Also induced were genes for several cell-wall localized heat-shock proteins – *HSP150* and *SSA2*. Along with upregulation of genes for structural cell wall proteins we observed upregulation of genes coding for some enzymes potentially involved in cell-wall restructuring and ECM formation, e.g., *SCW10* family 17 glucosidase (7-fold), *SIM1* glucosidase (6-fold), endo-1,3(4) beta glucanase *ENG1* (6-fold). Some structural and enzymatic cell wall components were downregulated. Strongly downregulatåd was the mannoprotein gene *DAN4* (3-fold) known to be repressed under aerobic growth conditions (Mrsa et al., 1999). These observations show that biofilm formation was accompanied by notable reorganization of the flor yeast cell wall.

**FIGURE 4:**
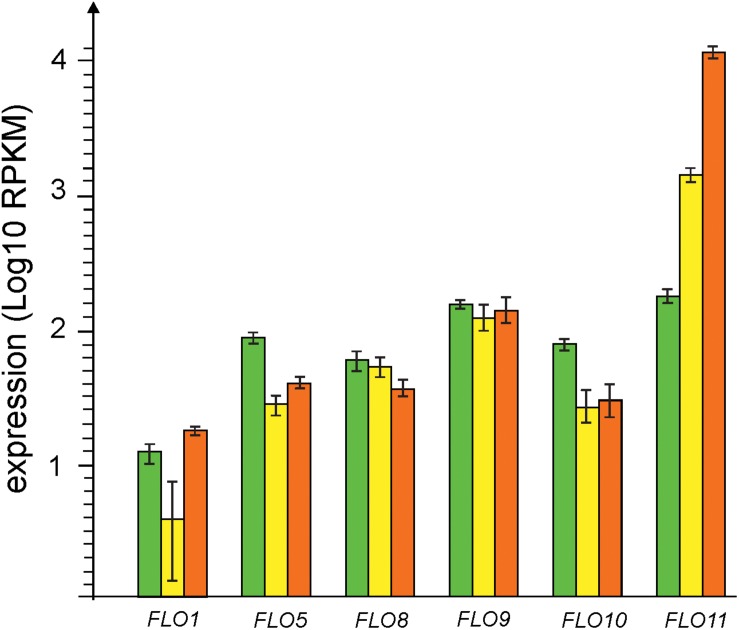
Changes in expression of genes of flocculins, – *FLO1* (gene 4857), *FLO5* (gene 3725), *FLO8* (gene 2945), *FLO9* (gene 5324), *FLO10* (gene 1874), and *FLO11* (gene 5300). Transcription levels observed at the stages of early, thin and mature biofilms are shown by green, yellow and red bars, respectively.

Expression of genes coding for 6 types of flocculins was detected at all three stages of biofilm development (Figure 4). At the early biofilm stage expression levels of all flocculins were comparable but at later stages all flocculin genes except *FLO11* were moderately down-regulated (*FLO5, FLO8*, and *FLO10*) or did not significantly changed their level of transcription (*FLO1* and *FLO9*). On the contrary, gene coding for Flo11-like adhesin was strongly up-regulated and its expression level reached maximum at the mature biofilm stage, accounting for more than 99% of all flocculins transcripts. Notably, *FLO11* was the fourth most highly expressed gene in the mature biofilm. Thus, these results show that Flo11 flocculin plays a key role in flor growth and maturation.

Increased requirements for structural and enzymatic cell wall components at the biofilm stage suggests upregulation of the major pathways for protein secretion and export. In the secretory pathway we observed upregulation of the genes for components of SEC61 translocon, the Srp14, Srp102 components of the signal recognition particle and signal peptidase, and Kar2 - the endoplasmic reticulum (ER) chaperone and unfolded protein response (UPR) regulator. Meanwhile, expression of genes for proteins involved in vesicle targeting, fusion and endocytosis did not show obvious alterations, majority of these genes were expressed at similar levels at the two compared stages (Supplementary Table S12).

### Endoplasmic Reticulum and Cytosolic Protein Quality Control

High ethanol and acetaldehyde concentrations in the course of biological wine aging impose significant burden on flor yeast physiology and metabolism and require activation of cellular defense mechanisms to ensure resistance and survival (Martínez et al., 1997; Esteve-Zarzoso et al., 2001; Castrejón et al., 2002; Aranda and del Olmo, 2003; Alexandre, 2013; Fierro-Risco et al., 2013). Under conditions of our experiment ethanol concentration was high already at the early biofilm stage and than slightly decreased, while concentration of acetaldehyde increased almost twice (Table 1).

Acetaldehyde is a highly reactive toxic compound for all organisms. Acetaldehyde-protein and acetaldehyde-DNA adducts cause conformational changes and inactivation of celluar targets, stoping cellular growth and inhibiting various cellular metabolic activities (Jones, 1990). Acetaldehyde-mediated accumulation of unfolded/misfolded proteins in ER may induce ER stress and trigger UPR as adaptive response to maintain cellular proteostasis (Ji, 2012). UPR is a complex interplay of activities like chaperones, glycosylation enzymes, trafficking pathways, endoplasmic-reticulum-associated protein degradation (ERAD) components etc, regulating the balance between protein synthesis, degradation and export and inducing apoptosis in conditions of unreleived stress (Cao and Kaufman, 2012).

Among the genes required for ER protein quality control most significantly upregulated were various chaperones, but not the ER ubiquitin ligases. Regarding ubiquitin-proteasomal system we found that many structural and regulatory proteasome subunit genes (*RPN1, RPN2, RNP10, RPN11, PRE6, PRE7* etc.) were upregulated at the mature biofilm stage (Supplementary Table S12). Thus, our gene expression analysis suggests that at the mature biofilm stage flor yeast cells do not experience more pronounced UPR stress relative to the early biofilm stage, but do induce the ERAD pathway as reflected by notable upregulation of the proteasomal genes and ER chaperone genes.

Yeast transcriptional responses to acetaldehyde exposure have been the subject of several studies (Aranda et al., 2002; Aranda and del Olmo, 2003, 2004). Under conditions of short-term acetaldehyde stress in the lab strain (Aranda and del Olmo, 2004) upregulated were genes for heat-shock proteins, sulfur metabolism, polyamine transporters, mediated by Met4 and Haa1 transcription factors, while downregulated were many genes involved in cellular division and cell-cycle progression. We have found that 75% of genes described as acetaldehyde-inducble by Aranda and del Olmo (2004) were also upregulated in our experimental conditions at mature biofilm stage (Supplementary Table S14). This common list includes *HSP* and *MET* genes, but not *TPO* genes for polyamine transporters. Upregulation of *MET* genes may be required for acetaldehyde detoxification through the synthesis of non-toxic compound.

### Autophagy and Cell Death

The specific environment of biological wine aging is characterized by nutrient starvation – a condition, potentially capable to induce both for authophagy and apoptosis. Evaluation of the known genes involved in micro and macro autophagy in yeast revealed that most of them were down-regulated at the mature biofilm stage. Notably, this downregulation was accompanied by a strong upregulation of *PCL5*, coding for a cyclin that in complex with Pho80 is known to negatively regulate autophagy (Measday et al., 1997). In contrast, many genes, known to be involved in apoptosis in yeast, were upregulated at this stage (Supplementary Table S12). These findings indicate opposing roles of autophagy and apoptosis in course of biofilm development.

Gene expression comparison revealed no clear pattern of up- or downregulation of genes involved in mitophagy. Most of mitophagy-related genes showed minor alterations in expression levels (Supplementary Table S12). Some positive mitophagy regulators, like *MDM34* and *ATG33* were upregulated and some negative regulators (*TOR1*, *TOR2*, *PTC6*) were downregulated. On the other hand, some important mitophagy mediators, mitophagy receptor *ATG32* and *MMM1* subunit of ERMES complex, were downregulated (Supplementary Table S12).

Apoptosis induction was accompanied by upregulation of the TF known to control apoptotic processes in yeast. Analysis of expression level of various vacuolar peptidases and nucleases indicated that apoptotic process is accompanied by extensive degradation of intracellular molecules and autolysis (Carmona-Gutierrez et al., 2010). Downregulation of many genes involved in peroxisome biogenesis in combination with downregulation of fatty acid and steroid biosynthetic processes suggests that fatty acid catabolism is suppressed at biofilm stage (Supplementary Table S15).

### Transport Protein Genes

There are almost 300 genes for established or predicted transmembrane transporters in *S. cerevisiae* genome, encoding proteins that facilitate the transport of a very wide variety of small compounds across the plasma and internal membranes, - amino acid, sugars, ions etc, that are differentially regulated depending on metabolic and physiological status of yeast cells, in nutrient-rich and starvation condition etc.

Yeast transport protein database (Brohée et al., 2010) is a resource dedicated to the precise classification and annotation of yeast transport protein genes (YTP genes). Of the 303 YTP genes stored at YTP database 287 were found in our RNA-seq data. For these genes we have compared the expression levels at different biofilm growth stages (Supplementary Table S16).

We observed sharp differences in YTP genes expression levels between the early and mature biofilm stages. Not surprisingly, at the mature biofilm stage we observed significant down-regulation of the majority of transporter genes. In particular, low-affinity glucose transporters (*HXT1* and *HXT3)*, oligopeptide (*OPT1,2*), allantoate (*DAL5*), polyamine (*TPO2,4*), amino acid (*PUT4, GAP1, BAT1, VBA1*), iron (*FET4*) transporters as well as ion pumps (*PMA1*) were downregulated.

However about 25% of genes from the analyzed set did not show notable alterations in expression levels between two stages and 10% showed notable up-regulation at the mature biofilm stage. Upregulated genes include high affinity hexose transporters (*HXT6, HXT7, HXT13*), enabling efficient uptake of sugars at low concentrations, *SSU1* sulfite transporter, *SUL2* sulfate transporter, *ENB1* iron transporter, several mitochondrial transporters for carboxylic acids (*MPC1, MPC2, SFC1*). The highest expression level at this stage was detected for *PIC2* gene encoding mitochondrial copper and phosphate carrier.

### “Uncharacterized” Genes Exhibiting Abundant Transcription and Significant Up and Down-Regulation

Despite consolidated community efforts in yeast comparative and functional genomics, functions of a significant number of yeast genes still remains unknown. According to recent SGD statistics (May 12, 2019), 1785 genes of *S. cerevisiae* in “Biological Process,” 1306 in “Cellular component” and 2562 in “Molecular Function” GO categories are deemed “unknown.” This uncertainty may be explained in part by the lack of information about specific conditions when these genes are actually needed. We reasoned that at least some of these genes may have an adaptive role under specific conditions of biological wine aging and biofilm formation.

In the Up4 list of genes highly up-regulated at the mature biofilm stage we found 19 genes within the category “protein of unknown function” (Supplementary Table S17). Several genes in this list overlap with the set of genes highly expressed in the mature biofilm. Search through SGD provided limited information concerning phenotypes conferred by null-mutants of these genes – majority fall in the category “competitive fitness – decreased” and “resistance to chemical – decreased.” We therefore manually searched the SPELL database^2^ (Hibbs et al., 2007) to identify conditions when these genes were considerably induced. Search terms were limited to various stress conditions, starvation, carbon and nitrogen metabolism that physiologically may resemble the flor yeast biofilm stage (Supplementary Table S18).

For the 9 selected up-regulated genes we found 21 studies when these genes were induced in a similar way as in our experiments. The most abundant categories were “stationary phase maintenance” (1 study, 8 genes), heat shock (5 genes, 6 studies), osmotic stress (4 genes, 5 studies), oxidative stress (6 genes, 4 studies), unfolded protein response (3 genes, 3 studies), starvation (4 genes, 3 studies), stress (5 genes, 3 studies). The genes most frequently detected in this search were *TMA10, UIP3, RTC3, RGI1*, and *YKR011C*. Of note, *TMA10, RTC3, RGI1*, along with *EGO4* were also among the top highly expressed genes at the mature biofilm stage. According to SGD description (Supplementary Table S17), *TMA10, RTC3, RGI1* are implicated in resistance to different forms of stresses, particularly, DNA replication stress and oxidative stress. The list of “unknown genes” among highly down-regulated is short; it involves four genes that in four studies were down-regulated in response to starvation and different stresses.

Of course, experimental conditions and strains genotypes utilized in our study and in those used for comparison were very different. Recent study concerning species-specific effects on stress induced gene expression show significant role of “biological noise” (Tirosh et al., 2011). Nevertheless, we consider that our comparison provides useful hints about the possible role of “unknown” genes at biofilm stage in flor yeast that can be further verified using various genome editing approaches.

### Sulfur Metabolism

*Saccharomyces cerevisiae* can use a variety of inorganic and organic sulfur sources that are taken up and assimilated through distinct transport and biochemical pathways (Thomas and Surdin-Kerjan, 1997). The major sulfur sources in wine materials and grape juice are sulfate (16–70 mg/l) and sulfite that is added to grape juice for wine making while the content of cysteine and methionine is rather low (<20 mg/l), so the sulfur assimilation pathway (SAP) is triggered during fermentation to support yeast growth (Huang et al., 2017). Biosynthesis of sulfur aminoacids in *S. cerevisiae* occurs through transsulfuration pathway, while in many other yeast species the alternative *O*-acetyl-serine (OAS) pathway also exists (Hébert et al., 2011).

We observed significant up-regulation of the genes of the SAP pathway in the samples derived from the mature biofilm as compared to the early biofilm stage. All three genes involved in first three steps of sulfate reduction to sulfite (Thomas and Surdin-Kerjan, 1997) - the *MET3* gene for ATP sulphurylase, the *MET14* gene for APS kinase, and the *MET16* gene for PAPS reductase were induced 3–4 fold. The *MET5* and *MET10* genes for subunits of sulfite reductase were also up-regulated 2–4 fold.

Coordinate up-regulation was also observed for many genes for initial steps in the cysteine and methionine biosynthesis pathways, mainly for *MET17* for bifunctional cysteine synthase/*O*-acetylhomoserine aminocarboxypropyltransferase responsible for sulfur incorporation into carbon chain, *MET2* for homoserine *O*-acetyltransferase, *CYS3* for cysthatione-gamma lyase. On the contrary, expression levels for genes for enzymes involved in methionine salvage pathway, for production of volatile sulfur compounds, and glutathione biosynthesis were not altered or down-regulated (Supplementary Table S19).

A very similar expression pattern for sulfur metabolism genes was observed in the study of transcription responses of laboratory strains to acetaldehyde exposure (Aranda and del Olmo, 2004). Authors showed that this induction is dependent upon transcriptional activators of the SAP and cysteine-methionine biosynthesis pathways. In our study we also observed up-regulation of genes for several relevant transactivators, namely for *MET4* (5-fold), *MET28* (6-fold), *MET32* (35-fold).

Observed induction of sulfur metabolism genes in the two studies may be relevant to protective yeast cell responses against toxic effects of acetaldehyde, for instance, through formation of non-toxic 1-hydroxyethane sulfonate in reaction between acetaldehyde and sulfite (Casalone et al., 1992). Contribution of other sulfur containing aminoacids and glutathione to these detoxifying effects is also plausible. Finally, in this context hydrogen sulfide should be considered not only as biosynthetic intermediate, but also as an important compound for yeast detoxification, population signaling, and life span extension (Hine et al., 2015).

### Genes Most Activated or Repressed at the Thin Biofilm Stage

As previously noted, for the most of DEGs, continuous up- or down-regulation was observed in course of flor growth. Nevertheless, 64 and 87 genes had, respectively, a maximum and a minimum of expression level at the intermediate (thin biofilm) stage (Supplementary Table S7).

The group of genes most highly expressed at the thin biofilm stage included amino acid (*BAP2*, *BAP3, DIP5, TAP1, TAT1*), iron (*FTR1*, *FET3*) and sulfate (*SUL1*) transporters, and plasma membrane permease *GIT1* mediating uptake of glycerophosphoinositol and glycerophosphocholine as sources of the inositol and phosphate. Up-regulation at this stage was also found for *GDH1* gene encoding glutamate dehydrogenase - the central enzyme in several nitrogen uptake pathways, *ARO10* gene encoding phenylpyruvate decarboxylase – the first enzyme in the Erlich amino acid degradation pathway, and *AAD14* gene for aryl-alcohol dehydrogenase. Up-regulation of these genes probably signaled metabolic shift of flor yeast toward utilization of specific nitrogen and carbon sources, available at this intermediate stage, that are apparently exhausted at the mature biofilm stage. Finally, up-regulation of genes for glutathione biosynthesis (*GSH2*, *GTT2*) and *YHB4* flavohemoglobin may indicate emergence of oxidative stress signal at this stage.

The group of genes down-regulated at the thin biofilm stage was included genes involved in abiotic stress response. In particular, genes involved in trehalose (*NTH1, TPS2, ATH1*) and glycerol (*GPD1, GPP2*) metabolism may mediate resistance to osmotic and ethanol stresses. Down-regulation of genes coding for some stress-related transcriptional factors (*MIG2, MIG3, MOT3, RSF2, USV1*), DNA damage-responsive protein (*DDR48*), other stress-related proteins (*GRE1*, *GRE3, GAD1*, *PAI3*, *SIP18*) suggested that some stresses are less pronounced at this stage.

Comparison of the lists of genes most strongly up- or down-regulated at the thin biofilm stage indicates that at the intermediate stage yeast cells were not completely limited in readily accessible carbon and nitrogen sources, and environmental conditions at this stage were to some extend less stressful than at the first and the last stages. Indeed, the early stage was characterized by adaptation to osmotic and ethanol stress, and the mature biofilm stage – to high acetaldehyde content, carbon and nitrogen starvation stress. Alterations in the expression of genes involved in amino acid and higher alcohol metabolism at the intermediate stage may influence production of aromatic compounds and contribute to final sensory compositions of wine.

## Discussion

Flor yeast strains are the soul of sherry wine formation (Peinado and Mauricio, 2009; Pozo-Bayón and Moreno-Arribas, 2011). Under typical sherry wine-making conditions flor yeast undergo important physiological, morphological and biochemical transitions to adapt to harsh oenological environment. Specific nutrient conditions – very low sugar and high ethanol content, high acetaldehyde concentration induce flor yeast to reprogram their metabolism from fermentative to oxidative one, and to withstand combined action of several stresses, namely nutrient limitation (especially for nitrogen), ethanol stress, oxidative stress, acetaldehyde stress and imbalance of nutritional agents (Alexandre, 2013; Moreno-García et al., 2016). In order to survive flor yeasts developed a biofilm (flor) – a specific multicellular aggregate that is widely considered as an adaptive mechanism ensuring oxygen access and promoting growth on non-fermentable carbon source (Zara et al., 2010).

Thorough characterization and quantification of accompanying transcriptional responses are critical for understanding the molecular mechanisms that govern these changes. To gain insight how gene expression variation contributes to changes in flor yeast physiology and metabolism under conditions of biological wine aging we have used RNA-seq technology to compare gene expression profiles of a flor yeast strain at different stages of biofilm growth. Application of RNA-seq platform enabled us to quantify expression levels of 5320 of 5323 genes annotated in the nuclear genome of flor yeast I-329 strain.

Our analysis showed significant transcriptome alteration with 816 genes up-regulated and 674 down-regulated (more than 2-fold) at the mature biofilm stage as compared to the early biofilm stage, reflecting metabolic and physiological changes in course of biofilm development. From the transcriptomic data the mature biofilm stage seems to resemble the quiescent stage with oxidative metabolism and activation of several stress resistance pathways. Indeed, nutritional conditions at the mature biofilm stage, tight cell–cell adhesion, toxic effects of ethanol and acetaldehyde should suppress cell proliferation, protein translation, and induce DNA damage (García-Ríos and Guillamón, 2019). However, continued expression of many genes required for transcription, translation, respiration, cell wall biogenesis etc suggests that at least some proportion of cells even in mature biofilm is in active, dividing state. In this regard it is necessary to point that flor yeast velum represents a rather heterogeneous multicellular structure composed of different yeast subpopulations thriving in various microenvironments. The difference of oxygen and nutrient availability, ethanol and acetaldehyde concentrations at the air-biofilm and biofilm-liquid interface may induce formation of various cell types that differ in cell metabolism etc. Such variation may lead to ultimate changes in gene expression as shown for yeast subpopulation within colonies grown on solid surfaces (Maršíková et al., 2017). Thus, whole-velum transcriptome data may in fact represent a superposition of transcriptomes of various cell subpopulations, differing in metabolic and physiological microenvironment. Nevertheless, very distinct and profound transcriptome upregulation at the mature biofilm stage was found for many genes, implicated in cell wall biogenesis and resistance to oxidative stress. For cell wall protein genes opposing trends in expression of *FLO11, HSP150, CIS3, PIR1* (upregulated) and *FLO5, FLO8*, *FLO10, DAN4* (downregulated) may reflect significant cell-wall remodeling in the course of flor formation and growth.

Several genes found among top highly expressed and upregulated genes at the mature biofilm stage, as for instance, *EGO4* and *RGI1*, encode proteins with “unknown” or poorly characterized function. Comparative analysis of the expression patterns of selected “unknown” genes with highest expression level using SPELL database indicated their upregulation at various stress conditions suggesting their unexplored adaptive role at the biofilm stage.

It is well known and shown in numerous studies using both microarray and RNA-seq platforms that transcriptional responses of yeast strains to different environmental cues are highly variable depending on the nature of abusing agent, time of exposure, strain genotype etc. (Taymaz-Nikerel et al., 2016; Ibáñez et al., 2017; Mendes et al., 2017; Tondini et al., 2019). It is also necessary to distinguish short and long-term responses and acquired resistance as mechanisms of adaptation (Stanley et al., 2010). The time scale of sampling points in these studies was in the range of hours and days, none had considered time points separated by several weeks. To the best of our knowledge this study is the first attempt to investigate transcriptome state along different stage of the development of the velum during biological aging. Flor yeast biofilms are typical sessile surface associated microbial populations, protected by polymeric extracellular matrix (ECM) (Zara et al., 2009; Costa-Orlandi et al., 2017). Biofilm formation is a gradual process, involving substrate adherence (flotation in the case of flor yeast), yeast cell proliferation over surface, cell-cell cohesion and embedment in ECM (Verstrepen and Klis, 2006). As shown in many studies with yeast and fungal human and plant pathogens, like *Candida albicans*, *Aspergillus fumigatus*, biofilm cells are phenotypically different from free or planktonic cells, are more resistant to environmental stresses and antifungals, that is a very important factor in their virulence (Fanning and Mitchell, 2012; Costa-Orlandi et al., 2017).

Transcriptome analysis of *C. albicans* and *A. fumigatus*, as well as *S. cerevisiae* during filamentous growth (Verstrepen and Klis, 2006), have revealed major alterations in gene expression patterns between biofilm cells and planktonic cells (García-Sánchez et al., 2004; Verstrepen and Klis, 2006; Gibbons et al., 2012). Despite obvious significant taxonomic differences between flor yeast and these pathogens, we found several common differential gene expression patterns pointing to existence of general mechanisms of transcriptional regulation in the course of biofilm development in these species. First of all this considers upregulation of many cell wall biogenesis genes and adhesins, required both for cell wall and ECM formation. Many of these genes are induced by Ace2 and Swi5 transcription factors, and, similar to *C. albicans*, we observe upregulation of these TF at mature biofilm stage.

Elevated expression of some genes, related to transcription, protein biosynthesis and turnover (ribosomal proteins, proteasomal genes, transcription and translation factors) is another common feature of flor yeasts and *C. albicans* and *A. fumigatus* biofilm cells (Fanning and Mitchell, 2012). This indicates that yeast and fungal biofilm cell are metabolically not dormant. In line with this observation was common upregulation of some amino acid biosynthesis genes, especially for sulfur aminoacids, permeases for poor nitrogen substrates in flor yeast and *C. albicans* biofilm cells (García-Sánchez et al., 2004).

In conclusion, important changes of gene expression of flor yeast strain I-329 at different winemaking stages were discovered as adaptive processes to biofilm formation. The observed differential expression patterns may reflect the physiologically and metabolically stable quiescent phase where different cell subpopulations had already gained resistance to specific nutrient conditions. The identification and functional analysis of biofilm-response genes could shed light on the overall understanding of cell response elicited by velum formation and sherry wine manufacturing conditions, and for the comprehension of relevant regulatory mechanisms.

## Data Availability Statement

The datasets generated for this study can be found in the SRR10551665, SRR10551664, SRR10551663, SRR10551662, SRR10551661, SRR10551660, SRR10551659, SRR10551658, SRR10551657.

## Author Contributions

AM, ME, and NR designed the research project and wrote the manuscript. SK, TT, AB, and AM performed the research. AB, AM, NR, and ME analyzed the data. All authors read and approved the manuscript.

## Conflict of Interest

The authors declare that the research was conducted in the absence of any commercial or financial relationships that could be construed as a potential conflict of interest.

## Footnotes

1 https://www.yeastgenome.org/locus/S000004600#paragraph

2 https://spell.yeastgenome.org/
