# Supplementary material for: Transcriptome Profile of Yeast Strain Used for Biological Wine Aging Revealed Dynamic Changes of Gene Expression in Course of Flor Development
Source: Front Microbiol. 2020 Apr 3;11:538. doi: 10.3389/fmicb.2020.00538 (PMC7145950; doi:10.3389/fmicb.2020.00538)
Supplement: Supplementary file 1 [file Data_Sheet_1.ZIP › Figure S1.pdf]

Early biofilm (sample 1)

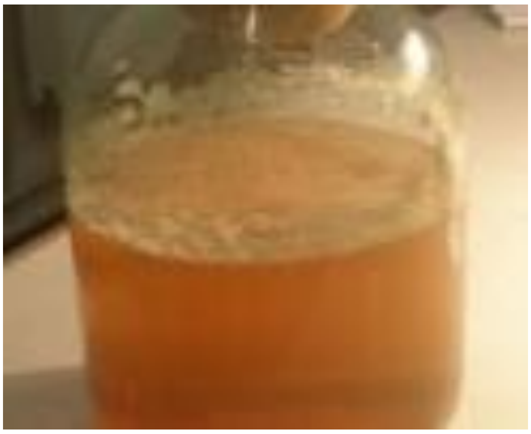

Thin biofilm (sample 2)

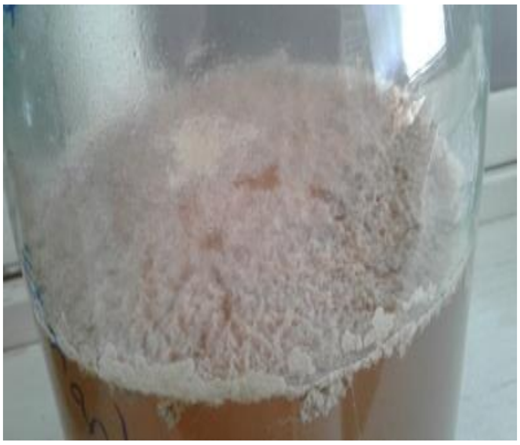

Mature biofilm (sample 3)

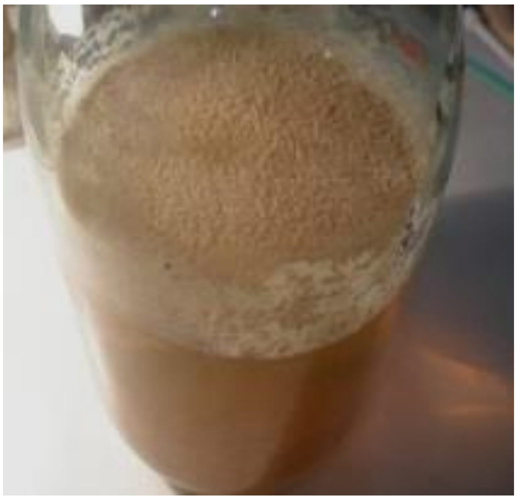

Figure S1. Images of flor yeast velum
