# Supplementary material for: Transcriptome Profile of Yeast Strain Used for Biological Wine Aging Revealed Dynamic Changes of Gene Expression in Course of Flor Development
Source: Front Microbiol. 2020 Apr 3;11:538. doi: 10.3389/fmicb.2020.00538 (PMC7145950; doi:10.3389/fmicb.2020.00538)
Supplement: Supplementary file 1 [file Data_Sheet_1.ZIP › Table S1.doc]

**Table S1**. Sequencing statistics.

| Stage | Sample ID | RNA-Seq reads | SRA accession |
| --- | --- | --- | --- |
| Early biofilm | IL20 | 7,5280,54 | SRR10551665 |
|  | IL21 | 6,897,699 | SRR10551664 |
|  | IL22 | 6,734,945 | SRR10551663 |
| Thin biofilm | IL23 | 6,309,292 | SRR10551662 |
|  | IL24 | 6,089,675 | SRR10551661 |
|  | IL25 | 5,980,833 | SRR10551660 |
| Mature biofilm | IL29 | 8,765,210 | SRR10551659 |
|  | IL30 | 6,912,070 | SRR10551658 |
|  | IL31 | 9,030,851 | SRR10551657 |
