# Supplementary material for: Transcriptome Profile of Yeast Strain Used for Biological Wine Aging Revealed Dynamic Changes of Gene Expression in Course of Flor Development
Source: Front Microbiol. 2020 Apr 3;11:538. doi: 10.3389/fmicb.2020.00538 (PMC7145950; doi:10.3389/fmicb.2020.00538)
Supplement: Supplementary file 1 [file Data_Sheet_1.ZIP › Table_S12.doc]

**Table S12**. Changes of expression of genes, involved in proteasomal system, UPR response and ERAD pathway, autophagy, mitophagy, DNA replication and repair, ribosome biogenesis, and transcription.

Mature biofilm and early biofilm stages has been compared

**Proteasome**

| **Category** | **Number** | **Genes** |
| --- | --- | --- |
| Up | 32 | PRE1 RPN9 SCL1 RPN2 PRE6 RPT2 RPT5 RPN6 PRE5 RPN13 PRE8 UMP1 RPN3 PUP1 PRE4 PRE3 RPN5 PRE7 RPT3 RPN11 RPN10 RPT6 RPN12 RPT4 PUP2 RPN1 RPN7 PUP3 PRE2 RPN8 PRE9 SEM1 |
| No change | 2 | RPT1 PRE10 |
| Down | 1 | BLM10 |

**Protein export**

| **Category** | **Number** | **Genes** |
| --- | --- | --- |
| Up | 8 | SPC2 SRP14 KAR2 SSS1 OXA1 SBH2 SRP102 SEC61 |
| No change | 13 | SBH1 SEC62 SEC65 SPC3 SEC63 SRP68 SRP54 SRP101 SEC11 IMP2 IMP1 SRP72 SSH1 |
| Down | 1 | SPC1 |

**Vesicle fusion**

| **Category** | **Number** | **Genes** |
| --- | --- | --- |
| Up | 4 | SED5 SNC2 SEC9 UFE1 |
| No change | 19 | GOS1 VPS45 VTI1 SEC17 SPO20 NYV1 TLG2 SSO1 YKT6 SEC22 VAM3 SNC1 USE1 BOS1 TLG1 SEC20 SFT1 MSO1 BET1 |
| Down | 1 | VAM7 |

**UPR and ERAD**

| **Category** | **Number** | **Genes** |
| --- | --- | --- |
| Up | 27 | SBH1 PDI1 SSE2 SSA2 HSP42 SSE1 DSK2 UFD1 FES1 KAR2 YET3 SSA4 JEM1 MPD1 UBC4 CDC48 HSC82 SHP1 HSP82 LHS1 RAD23 SSS1 SBH2 DFM1 HSP26 SEC61 SSA1 |
| No change | 46 | SSM4 SEC62 UBC7 EUG1 OST1 SUI2 DOA1 SSH1 SEC24 STT3 SEC63 MNL2 SNL1 YDJ1 SSB2 EMP46 PNG1 HRD1 OST3 SEC31 SCJ1 SSA3 HRD3 CDC53 SIL1 SWP1 UBC5 SEC13 SAR1 EPS1 SKP1 YOS9 DER1 SFB3 UBX2 EMP47 OST5 HRT1 WBP1 GCN2 MNS1 SEC23 ROT2 CUE1 HLJ1 UBC6 |
| Down | 13 | UFD2 SED4 USA1 CNE1 IRE1 SFB2 KRE5 OTU1 OST6 SEC12 CWH41 GTB1 NPL4 |

**Autophagy**

| **Category** | **Number** | **Genes** |
| --- | --- | --- |
| Up | 15 | VTI1 VAM3 TAP42 TIP41 LCB1 VPS3 ARG82 PEP4 ATG8 ARP2 SNF1 ATG16 GCN4 PCL5 RAS2 |
| No change | 38 | UME6 PPH21 YKT6 MON1 VPS30 ATG4 YPT7 SUI2 LCB2 ARP3 ATG3 ATG11 SEC17 ATG14 PRB1 VPS41 ATG13 ATG31 VPS45 ATG12 ATG27 ATG23 SLT2 PEP3 ATG21 TPK1 TOS3 ATG17 ATG1 RAS1 VAM6 AVT3 TPK3 LST8 GCN2 ATG10 CCZ1 ATG29 |
| Down | 31 | PEP5 SAK1 KOG1 KCS1 ATG5 YSP3 SEC18 PHO85 ATG7 MSN2 TPK2 YMR1 VAM7 TOR2 VPS33 ATG18 ELM1 VPS8 AVT4 ATG22 VPS15 VPS34 ATG2 ATG15 TOR1 RIM15 MSN4 VPS16 SCH9 PPH22 ATG9 |

Mitophagy

| **Category** | **Number** | **Genes** |
| --- | --- | --- |
| Up | 7 | BRE5 FIS1 PAF1 MDM38 FMC1 MDM34 ATG33 |
| No change | 29 | ATG8 CKA1 HOG1 CTR9 UBP3 BCK1 CKA2 CKB1 CKB2 DNM1 MDM12 MKK1 MKK2 PBS2 SIN3 SLT2 SMD3 SSK1 UME6 VPS1 WSC2 WSC3 ATG1 MSS4 ATG11 MDM10 MIP1 YME1 SLG1 |
| Down | 7 | PKC1 ATG32 SLN1 TOR2 MMM1 TOR1 PTC6 |

**DS Break Repair**

| **Category** | **Number** | **Genes** |
| --- | --- | --- |
| Up | 5 | POL32 RDH54 RAD59 SEM1 RAD54 |
| No change | 13 | MRE11 MUS81 RAD55 RAD57 RAD50 RAD52 TOP3 SGS1 RFA1 RFA2 RAD51 POL31 XRS2 |
| Down | 2 | TEL1 POL3 |

**DNA replication**

| **Category** | **Number** | **Genes** |
| --- | --- | --- |
| Up | 2 | POL32 POL30 |
| No change | 25 | RFC3 RFC2 RFC1 RAD27 MCM2 POL2 PRI1 RFC4 DPB2 POL12 POL1 RFC5 DNA2 RNH1 DPB3 MCM5 RFA1 RNH201 RFA2 DPB4 MCM7 MCM6 POL31 CDC9 DLS1 |
| Down | 4 | MCM4 MCM3 PRI2 POL3 |

**Mismatch Repair**

| **Category** | **Number** | **Genes** |
| --- | --- | --- |
| Up | 5 | POL32 EXO1 SRS2 POL30 MSH3 |
| No change | 14 | RFC3 RFC2 RFC1 MLH1 RFC4 RFC5 MLH3 MSH6 MSH2 RFA1 RFA2 PMS1 POL31 CDC9 |
| Down | 1 | POL3 |

**Nucleotide Excision Repair**

| **Category** | **Number** | **Genes** |
| --- | --- | --- |
| Up | 6 | *POL30 TFB3 POL32 CCL1 RAD23 SRS2* |
| No change | 24 | *RFC3 SSL2 RAD28 POL2 KIN28 RFC5 RFA2 DPB4 DLS1 RAD2 RFC1 RFC2 TFB1 RAD3 HRT1 TFB2 RFC4 DPB2 DPB3 RFA1 TFB5 RAD14 POL31 CDC9* |
| Down | 6 | *SSL1 RAD26 POL3 TFB4 RAD4 RAD1* |

**Ribosome**

| **Category** | **Number** | **Genes** |
| --- | --- | --- |
| Up | 73 | RPL10 RSM18 RPS22a RPS28b MRPS5 RPL14b RLP24 RPL34a RPP1b RPS19b RPS25a RPP2a RSM10 RPS9b RPL27a RPS17b RPS18b MRPS28 RPS24b RPS12 MRPS17 RPL22a RPL12a RPL24b RPP2b RPL6a RPS10a RPP0 MRPL11 RPS10b RPL37a RPS3 RPS16a RPL7b MRPS12 MRPL32 RPL24a RPL43a MNP1 RPS19a RPL31a RPL34b MRP17 MRPL9 RPL38 RPS21b RPL18a RPL32 RPS26a RPS11a MRPL7 RPL36b RPL21b RPS20 MRP20 MRPS16 MRPL40 RPL33a RPL11b RPS13 RLP7 RPS21a RPS26b RPL35b RPL37b RPS31 MRP2 RPS28a RSM7 RPP1a MRPL24 RPS7b RPS15 |
| No change | 56 | MRPL23 RPL33b RPL17b RPL40b RPL15b RPL13b RPL9b MRPS9 RPS2 RPL21a RPL16a RPL7a RPL2b MRPL10 RPL13a RPL28 RPL16b RPS6a RPL1a RPL3 RPS5 RPS8b RPL30 RPL25 RPS27a RPL11a RPL4b MRPL6 RPS7a RPL19a RPL42a RPL20b RPS22b RPL17a RPL26b RPS25b RPS14a RPS14b RPL22b RPS1a MRP4 RPS17a RPS23b RPL15a MRPS18 RPS4a RPL8b RPL6b MRPL19 RPL4a RPL5 RPS0a RPL23b YML6 RPS1b RPL31b |
| Down | 3 | RPS0b RPL8a RPS9a |

**Ribosomal biogenesis**

| **Category** | **Number** | **Genes** |
| --- | --- | --- |
| Up | 17 | RNH70 RIO1 CKA1 SNU13 GSP2 FCF1 RIX7 NOP56 POP6 CBF5 NHP2 UTP9 NOP58 GSP1 XRN1 POP4 MTR2 |
| No change | 46 | RAT1 UTP13 CKA2 NOP4 LSG1 EMG1 UTP6 POP3 BMS1 TIF6 UTP15 UTP8 UTP18 DIP2 RRP7 MEX67 RNT1 IMP3 UTP4 NUG1 POP8 IMP4 UTP5 REX2 NMD3 PWP2 UTP21 KRE33 POP1 SNM1 HRR25 CRM1 MDN1 GAR1 POP5 SDO1 NOP1 NOB1 RCL1 RMP1 RPP1 UTP14 CKB2 CKB1 FAP7 RIO2 |
| Down | 8 | NAN1 UTP10 MPP10 RIA1 NOG1 NOG2 AFG2 UTP22 |

**RNA polymerase**

| **Category** | **Number** | **Genes** |
| --- | --- | --- |
| Up | 8 | RPB4 RPB11 RPC31 RPA14 RPB10 RPA34 RPC19 RPB5 |
| No change | 19 | RPC53 RPA12 RPC40 RPC25 RPB2 RPC37 RPC10 RPC82 RPB3 RPA49 RPO21 RPB7 RPO26 RPA135 RPC11 RPC34 RPB8 RPA43 RET1 |
| Down | 2 | RPO31 RPA190 |

Basal TF

| **Category** | **Number** | **Genes** |
| --- | --- | --- |
| Up | 12 | CCL1 TFG1 TAF10 TAF8 TAF14 NPL3 TAF12 SPT15 TAF5 TOA1 TAF7 TFB3 |
| No change | 16 | TAF1 TFB1 TAF11 RAD3 TFA1 KIN28 TFB2 TFA2 TAF13 TAF3 TOA2 TFB5 TAF9 TFG2 SUA7 SSL2 |
| Down | 3 | TAF2 TAF6 SSL1 |

| **Category** | **Names** | **Genes** |
| --- | --- | --- |
| Up | 20 | YHP1 DBF2 CYC8 PCL1 CKS1 GIN4 CDC5 SPO12 SLK19 SIC1 CDC20 YOX1 HSL1 MAD1 SWI5 CLN2 CLN1 MCD1 DUN1 RFX1 |
| No change | 87 | APC4 PHO5 PHO2 DBF20 CDC16 SWI4 MRC1 MIH1 PDS1 CDC15 BUB1 PPH21 ORC4 CDH1 BUB2 YCS4 YCG1 ORC3 WHI5 MCM5 CLB6 SMC1 CDC6 CDC28 MCM6 CLB5 LTE1 BUB3 GRR1 CAK1 TUP1 MAD2 MCM2 SMC3 APC5 ORC2 MOB1 HSL7 KCC4 MEC3 DAM1 CHK1 SCC2 APC9 MCM1 SMC4 BRN1 TAH11 FUS3 RAD9 DBF4 MPS1 IRR1 CDC7 CLB2 SMC2 TEM1 PHO4 CDC53 CDC4 RAD53 FAR1 CDC23 CDC55 SKP1 CDC14 APC2 CDC26 RAD24 APC11 MBP1 SWM1 SWI6 HRT1 PCL2 SWE1 CLB3 SCC4 RAD17 DDC1 PHO80 CDC45 BFA1 NET1 MCM7 CLB4 CLB1 |
| Down | 19 | PHO81 MET30 MEC1 MCM3 FOB1 TPD3 PHO85 CDC27 ORC1 ESP1 MAD3 MCM4 CLN3 LCD1 ORC6 ORC5 APC1 PPH22 DOC1 |
