# Supplementary material for: Transcriptome Profile of Yeast Strain Used for Biological Wine Aging Revealed Dynamic Changes of Gene Expression in Course of Flor Development
Source: Front Microbiol. 2020 Apr 3;11:538. doi: 10.3389/fmicb.2020.00538 (PMC7145950; doi:10.3389/fmicb.2020.00538)
Supplement: Supplementary file 1 [file Data_Sheet_1.ZIP › Table_S18.doc]

# **Table S18**. “Uncharacterized” genes upregulated at the mature biofilm stage were also upregulated under related physiological conditions.

References are numeric. The intensity of grey colour in the cells is proportional to the number of studies revealed.

| SPELL database terms | YNR071C | EGO4 | TMA10 | CSS3 | RTC3 | UIP3 | RGI1 | YKR075C | YKR011C |
| --- | --- | --- | --- | --- | --- | --- | --- | --- | --- |
| Stationary phase maintenance | [1] |  | [1] | [1] | [1] | [1] | [1] | [1] | [1] |
| Organelles, biogenesis, structure, function |  |  |  |  |  |  |  |  |  |
| Carbon utilization |  | [2] | [3] |  |  |  |  |  |  |
| Cell wall organization |  |  |  |  |  |  |  |  |  |
| Cellular ion homeostasis |  |  |  |  | [4] |  |  |  |  |
| DNA damage stimulus |  |  | [1] |  |  |  |  |  |  |
| filamentous growth | [5] |  |  |  |  | [5] |  |  |  |
| flocullation |  |  |  |  |  |  |  |  |  |
| heat shock | [1] |  | [1,6,7] | [1] | [1,8] |  | [1,7–9] |  | [1,6,8] |
| nitrogen utilization |  | [10] |  |  |  |  |  |  |  |
| osmotic stress |  | [10] | [6,10,11] |  |  | [1,6,11] | [10] |  | [6,10] |
| oxidative stress | [1] |  | [6] | [1] | [1,12] | [13] |  |  | [1,13] |
| unfolded protein response |  |  | [14,15] |  | [16] |  |  |  | [1] |
| starvation |  |  | [3,6,11] |  | [3,11] | [11] |  |  | [11] |
| stress |  | [17] | [18] |  | [19] |  | [19,20] | [9] |  |
| diauxic shift |  |  | [21] |  | [21] | [11] | [2] |  |  |

References

1. Gasch A.P. et al. Genomic expression programs in the response of yeast cells to environmental changes. // Mol. Biol. Cell. 2000. Vol. 11, № 12. P. 4241–4257.

2. Roberts G.G., Hudson A.P. Transcriptome profiling of Saccharomyces cerevisiae during a transition from fermentative to glycerol-based respiratory growth reveals extensive metabolic and structural remodeling // Mol. Genet. Genomics. 2006. Vol. 276, № 2. P. 170–186.

3. Chechik G. et al. Activity motifs reveal principles of timing in transcriptional control of the yeast metabolic network // Nat. Biotechnol. 2008. Vol. 26, № 11. P. 1251–1259.

4. Barreto L. et al. The short-term response of yeast to potassium starvation // Environ. Microbiol. 2012. Vol. 14, № 11. P. 3026–3042.

5. Prinz S. et al. Control of yeast filamentous-form growth by modules in an integrated molecular network // Genome Res. 2004. Vol. 14, № 3. P. 380–390.

6. Berry D.B., Gasch A.P. Stress-activated Genomic Expression Changes Serve a Preparative Role for Impending Stress in Yeast // Mol. Biol. Cell. 2008. Vol. 19, № 11. P. 4580–4587.

7. Düvel K. et al. Multiple roles of Tap42 in mediating rapamycin-induced transcriptional changes in yeast // Mol. Cell. 2003. Vol. 11, № 6. P. 1467–1478.

8. Segal E. et al. Module networks: Identifying regulatory modules and their condition-specific regulators from gene expression data // Nat. Genet. 2003. Vol. 34, № 2. P. 1667–176.

9. Pastor-Flores D. et al. Depletion of yeast PDK1 orthologs triggers a stress-like transcriptional response // BMC Genomics. 2015. Vol. 16. P. 719.

10. Chasman D. et al. Pathway connectivity and signaling coordination in the yeast stress-activated signaling network. // Mol. Syst. Biol. 2014. Vol. 10. P. 759.

11. Bradley P.H. et al. Coordinated concentration changes of transcripts and metabolites in Saccharomyces cerevisiae // PLoS Comput. Biol. 2009. Vol. 5, № 1. P. e1000270.

12. Shapira M. Disruption of Yeast Forkhead-associated Cell Cycle Transcription by Oxidative Stress // Mol. Biol. Cell. 2004. Vol. 15, № 12. P. 5659–5669.

13. Guan Q. et al. Cellular memory of acquired stress resistance in Saccharomyces cerevisiae // Genetics. 2012. Vol. 192, № 2. P. 495–505.

14. Oromendia A.B., Dodgson S.E., Amon A. Aneuploidy causes proteotoxic stress in yeast // Genes Dev. 2012. Vol. 26, № 24. P. 2696–2708.

15. Travers K.J. et al. Functional and genomic analyses reveal an essential coordination between the unfolded protein response and ER-associated degradation // Cell. 2000. Vol. 101, № 3. P. 249–258.

16. Levy S. et al. Strategy of transcription regulation in the budding yeast // PLoS One. 2007. Vol. 2, № 2. P. e250.

17. Lai L.-C. et al. Metabolic-State-Dependent Remodeling of the Transcriptome in Response to Anoxia and Subsequent Reoxygenation in Saccharomyces cerevisiae // Eukaryot. Cell. 2006. Vol. 5, № 9. P. 1468–1489.

18. Shivaswamy S., Iyer V.R. Stress-Dependent Dynamics of Global Chromatin Remodeling in Yeast: Dual Role for SWI/SNF in the Heat Shock Stress Response // Mol. Cell. Biol. 2008. Vol. 28, № 7. P. 2221–2234.

19. Lewis J.A. et al. Genetic Architecture of Ethanol-Responsive Transcriptome Variation in Saccharomyces cerevisiae Strains // Genetics. 2014. Vol. 198, № 1. P. 369–382.

20. Casamayor A. et al. The role of the Snf1 kinase in the adaptive response of Saccharomyces cerevisiae to alkaline pH stress // Biochem. J. 2012. Vol. 444, № 1. P. 39–49.

21. DeRisi J.L., Iyer V.R., Brown P.O. Exploring the metabolic and genetic control of gene expression on a genomic scale // Science. 1997. Vol. 278, № 5338. P. 680–686.
